# Plant Growth, Antibiotic Uptake, and Prevalence of Antibiotic Resistance in an Endophytic System of Pakchoi under Antibiotic Exposure

**DOI:** 10.3390/ijerph14111336

**Published:** 2017-11-03

**Authors:** Hao Zhang, Xunan Li, Qingxiang Yang, Linlin Sun, Xinxin Yang, Mingming Zhou, Rongzhen Deng, Linqian Bi

**Affiliations:** 1 School of Environment, Henan Normal University, Xinxiang 453007, China; kele1564@126.com; 2 College of Life Sciences, Henan Normal University, Xinxiang 453007, China; 15516551517@163.com (X.L.); nlycds@163.com (L.S.); 15736966832@163.com (X.Y.); zm921011@163.com (M.Z.); dengrongzhen127@163.com (R.D.); 15736927022@163.com (L.B.); 3 Key Laboratory for Microorganisms and Functional Molecules, University of Henan Province, Xinxiang 453007, China

**Keywords:** antibiotics, pakchoi, endophytic bacteria, antibiotic-resistant genes, hydroponic cultivation

## Abstract

Antibiotic contamination in agroecosystems may cause serious problems, such as the proliferation of various antibiotic resistant bacteria and the spreading of antibiotic resistance genes (ARGs) in the environment or even to human beings. However, it is unclear whether environmental antibiotics, antibiotic resistant bacteria, and ARGs can directly enter into, or occur in, the endophytic systems of plants exposed to pollutants. In this study, a hydroponic experiment exposing pakchoi (*Brassica chinensis* L.) to tetracycline, cephalexin, and sulfamethoxazole at 50% minimum inhibitory concentration (MIC) levels and MIC levels, respectively, was conducted to explore plant growth, antibiotic uptake, and the development of antibiotic resistance in endophytic systems. The three antibiotics promoted pakchoi growth at 50% MIC values. Target antibiotics at concentrations ranging from 6.9 to 48.1 µg·kg^−1^ were detected in the treated vegetables. Additionally, the rates of antibiotic-resistant endophytic bacteria to total cultivable endophytic bacteria significantly increased as the antibiotics accumulated in the plants. The detection and quantification of ARGs indicated that four types, *tet*X, *bla*_CTX-M_, and *sul*1 and *sul*2, which correspond to tetracycline, cephalexin, and sulfamethoxazole resistance, respectively, were present in the pakchoi endophytic system and increased with the antibiotic concentrations. The results highlight a potential risk of the development and spread of antibiotic resistance in vegetable endophytic systems.

## 1. Introduction

Antibiotic pollutants and their environmental impacts have become a mounting concern owing to their broad usage and persistence in the environment. A large range of veterinary and human antibiotics have been detected in soil, animal manure, sediment, municipal or industry wastewater, surface water, groundwater, and drinking water samples [1,2,3,4,5,6]. In agroecosystems, the contamination of various antibiotics, such as tetracyclines, sulfonamides, and fluoroquinolones, is a substantial problem globally, and especially in China [7,8,9]. A dominant source of agricultural antibiotic contamination is due to ~75% of the antibiotics ingested by animals passing unaltered through their digestive tracts, with the result that antibiotics are released in the field directly in feces or urine, or indirectly through the application of manure as fertilizer [10,11,12]. Another source is the irrigation of crops using wastewater containing antibiotics [13,14,15].

Once antibiotics are released into agricultural lands, crops are exposed to them due to their persistence, and the level of exposure depends on the physicochemical properties of the compounds, sorption potential, and environmental conditions [16,17]. Even if some antibiotics are degraded to a certain degree, most of them are replaced by ongoing use and release [18]. Under antibiotic contamination conditions, certain pharmaceutical compounds (such as tetracycline, oxytetracycline, sulfamethazine, sulfamethoxazole, tylosin, trimethoprim, ofloxacin, ciprofloxacin, and amoxicillin) can be absorbed by plants (such as wheat, corn, rice, lettuce, cabbage, spinach, carrot, cucumber, tomato, and potato) from the growth media through their roots and accumulate [13,14,15,19,20,21,22]. Although the human health implications of antibiotic pollutants in plant crops are largely unknown, several potential adverse impacts, including allergic reactions, chronic toxic effects as a result of prolonged exposure, and even the disruption of digestive system functions, have been speculated [16,23,24]. Thus, there is a growing concern that antibiotic pollution in food crops makes its way into food supply systems.

To date, the majority of research on the impact of antibiotic contamination in plants has focused on evaluating the toxicity of antibiotics to plants or detecting the ability of antibiotics to accumulate in plants. Limited knowledge is available regarding the potential effects of antibiotic stress on the development and spread of antibiotic resistance, including antibiotic-resistant bacteria and antibiotic-resistant genes (ARGs), in plant endophytic systems. There is a diverse range of endophytic bacteria, which includes pathogens, mutualists, and commensals that grow within the roots, vasculature, and aerial tissues of plants [25]. Recently, antibiotic resistance in endophytic bacteria isolated from medicinal plants has been reported [26,27]. Our previous research also reported a high prevalence of antibiotic-resistant endophytic bacteria (AREB), including some resistant to more than three different types of antibiotics, in various manure-fertilized vegetables, such as celery, pakchoi, and cucumber [28]. However, it is unclear whether the antibiotic resistance of endophytic bacteria can be impacted directly by antibiotic pollution in the environment, especially in the edible parts of vegetables.

To assess possible consequences, pakchoi (*Brassica chinensis* L.), a frequently consumed vegetable in China, was selected and planted in a hydroponic system and exposed to different antibiotics. Then, the antibiotic uptake and its effects on plant growth and the presence of AREB and ARGs in the endophytic system were investigated and evaluated. The findings will facilitate a more accurate assessment of the potential risks of antibiotic contamination to food quality and environmental health.

## 2. Materials and Methods

### 2.1. Chemicals and Reagents

Tetracycline (TC, >98.0%), cephalexin (CPL, >99.0%), and sulfamethoxazole (SMX, >99.5%) were purchased from Dr. Ehrenstorfer GmbH (Augsburg, Germany) and selected to represent the different classes of antibiotics (tetracyclines, β-lactams, and sulfonamides, respectively) based on their frequent usage in the local livestock farms in Xinxiang City, China [28]. Tetracyclines are broad-spectrum antibiotics that inhibit bacterial protein synthesis by preventing the attachment of aminoacyl-tRNA to the ribosomal acceptor (A) site. Resistance to tetracyclines has now emerged in many pathogenic bacteria due to genetic acquisition of *tet* genes, which include efflux genes, ribosomal protection genes, and enzymatic modification genes [29]. β-lactam antibiotics are the most widespread class of human antibacterials that inhibit bacteria by interfering with cell wall synthesis. The most major mechanism of bacterial resistance to β-lactam is the expression of β-lactamases that hydrolyze the antibiotic [30]. Sulfonamides, which are synthetic antibacterial drugs, inhibit bacterial folate biosynthesis by competing with the natural substrate *p*-amino-benzoic acid for binding to dihydropteroate synthase (DHPS), an enzyme in the folic acid synthesis pathway. Two genes, *sul*1 and *sul*2, mediated by transposons and plasmids, and expressing DHPS highly resistant to sulfonamide, have been found [31].

Methanol, acetonitrile, formic acid, and acetone of HPLC grade were purchased from Fisher Scientific (Fair Lawn, NJ, USA). Other chemicals were of analytical grade and obtained from Yaohua Chemical Reagent Factory (Tianjin, China). Ultrapure water was supplied using a Millipore Milli-Q system (Billerica, MA, USA). Oasis HLB cartridges (hydrophilic-lipophilic balance, 6 mL, 500 mg) purchased from Waters (Milford, MA, USA) were used for the extraction and purification of the target antibiotics. Individual stock standards were prepared by dissolving antibiotics separately in methanol and were stored at −20 °C in brown vials.

### 2.2. Hydroponic Experimental Procedure

A hydroponic experiment with antibiotic treatments was performed in an experimental greenhouse in the College of Life Sciences, Henan Normal University, China, during the autumn of 2016. Seeds of pakchoi (*B. chinensis* L.) were used for this study. TC, CPL, and SMX were separately added into the hydroponic solution of the test system at two concentrations: 50% of minimum inhibitory concentration (MIC) of each antibiotic, and the MIC of each antibiotic [32]. Each of the MIC values in the hydroponic solution was set as the induced dose of resistant bacteria.

Prior to testing, the seeds were surface-sterilized in 0.1% sodium hypochlorite solution for 10 min and then rinsed with sterile deionized water [33]. Seeds on a piece of sterile filter paper were placed into 10 cm sterile Petri dishes, and 10 mL of sterile water was added. Then, the Petri dishes were covered with their lids and maintained in a dark incubator at 25 ± 2 °C. After germination, the seeds were transferred to a plastic cuboid hydroponic tank (45 × 20 × 17 cm). All tanks were wiped with 75% ethanol and thoroughly rinsed with deionized water before first use. After that, each tank was filled with 12 L of Hoagland nutrient solution [34] or Hoagland nutrient solution supplemented with an antibiotic. The treatments were as follows: (1) control with no antibiotics added; (2) TC-treated (at concentrations of 8 and 16 mg·L^−1^, respectively); (3) CPL-treated (at concentrations of 32 and 64 mg·L^−1^, respectively); and (4) SMX-treated (at concentrations of 38 and 76 mg·L^−1^, respectively). Each treatment was designed with three replicates, and a total of 21 hydroponic tanks were used in the present study. Additionally, each tank had nine cylindrical holes (4 cm depth and 3 cm diameter) in its cover containing sponges (3 × 3 × 2.5 cm) in individual cylinders as a rooting medium. Five uniform seeds were planted per hole and irrigated with half-strength Hoagland solution every 2 days until the roots of the seedlings were immersed in the solution. Finally, only one or two strong seedlings were selected to leave in each hole, and they were grown directly in nutrient solution.

During the experiment the room conditions were maintained at 25 ± 2 °C in daylight and 18 ± 2 °C at night, with a relative humidity between 65% and 70%. Each planter was equipped with an electric aeration pump and was aerated for 2 h every day. Because of evapotranspiration from the vessels, lost water was supplemented with fresh nutrient solution without the addition of extra antibiotics. Pakchoi was harvested after 55 days of cultivation. Then, the sponges attached to the vegetables were trimmed off. The plants were rinsed first with tap water and then with deionized water and dried on adsorbent paper. The growth parameters and abundance of endophytic bacteria were measured immediately. Antibiotic analyses and DNA extraction from endophytic bacteria were completed within two weeks of sampling.

### 2.3. Measurements of Growth Parameters

All plants were harvested at the end of the test, and then, plant heights, root lengths, and fresh biomasses of 10 plants from each treatment were measured and recorded. The growth inhibition rate was calculated using following formula:

% inhibition = (M_0_ − M_t_)/M_0_ × 100

where M_0_ indicates the measurement of the control treatment and M_t_ indicates the measurement of the antibiotic treatment.

### 2.4. Antibiotic Analyses in Plants

Samples of the edible pakchoi portions (stem and leaf) were selected and freeze-dried for 24 h until moisture was no longer present [35]. Then, the freeze-dried samples were ground thoroughly, and the amount of antibiotics in the plant tissues were determined using ultrasonic extraction, solid-phase extraction, and liquid chromatography-mass spectrometry. The extraction method and clean-up procedure used was already described for the analysis of Chinese white cabbage, water spinach, and other crops [15]. Thereafter, the target compounds from treated samples were analyzed using an ultra-performance liquid chromatography-tandem mass spectrometer (Waters, Milford, MA, USA) equipped with an electrospray ionization source in multiple-reaction monitoring mode. Details of the quantitative analysis were described by Gros et al. [36].

### 2.5. Enumeration of Total Cultivable Endophytic Bacteria (TCEB) and AREB

To isolate endophytic bacteria, the edible portions of fresh pakchoi were immersed in 3% hydrogen peroxide for 30 min, followed by rinses with sterile deionized water (3 min × 3 times). Then, they were immersed in 70% ethanol for 1 min and rinsed as before [37]. Finally, surface-sterilized samples were dried using sterilized filter papers. To ensure the complete surface disinfection, 100 μL of the last wash water was spread on meat-peptone agar and cultivated at 30 °C for 3 days to check for colony growth [38]. Samples with no bacterial growth were considered successfully sterilized. For each experimental treatment, the disinfected vegetable was cut with a sterile scalpel into pieces and ground together with quartz sand in a sterile mortar. Then, 3 g of ground tissue was mixed with 10 mL of sterile water and the mixture was diluted to 10^−3^. Each 100 μL of diluted suspension was spread on meat-peptone agar and on corresponding antibiotic-containing agars (TC, CPL, and SMX at concentrations of 16, 64, and 76 mg·L^−1^, respectively) for cultivation at 28 °C for 3 days. Each sample was replicated three times. The colony-forming units (CFUs) of TCEB and AREB (endophytic bacteria resistant to TC, CPL, and SMX, respectively) were enumerated.

### 2.6. DNA Extraction, PCR Detection, and ARGs Quantification

The surface-sterilized edible pakchoi portions were cut into pieces and ground with liquid nitrogen before extraction under sterile conditions. Total DNA was extracted using PowerPlant DNA Isolation Kit (MoBio Laboratories, San Diego, CA, USA) following the manufacturer’s instructions [37]. The concentrations and qualities of the extracted DNA samples were determined using a NanoDrop ND-2000 spectrophotometer (Thermo Scientific, Waltham, MA, USA) and agarose gel electrophoresis, respectively.

PCR detection assays were used to screen for the presence or absence of 23 types of ARGs in the antibiotic-treated samples, including 12 tetracyclines-resistant genes (*tet*A, *tet*C, *tet*G, *tet*K, *tet*L, *tet*M, *tet*O, *tet*Q, *tet*T, *tet*W, *tet*B/P, and *tet*X), 5 sulfonamides-resistant genes (*sul*1, *sul*2, *sul*3, *dfr*A1, and *dfr*A7), and 6 β-lactams-resistant genes (*bla_amp_*_C_, *bla*_VIM_, *bla*_CTX-M_, *bla*_TEM_, *bla*_SHV_, and *bla*_Z_). PCR detection assays were performed as previously described [39]. Primers and annealing temperatures are described in Table S1.

The positive ARGs and eubacterial 16S rRNA gene were quantified by fluorescence quantitative PCR (qPCR) using a LightCycler real-time PCR system (Roche, Basel, Switzerland) with SYBR Green I. Details of the primers are listed in Table S2. Plasmids carrying target genes in the pMD19-T vector (TaKaRa, Ostu Shiga, Japan) were constructed to produce the standard curves [40], which consisted of at least five orders of magnitude (R^2^ > 0.99) (Table S3). The 20 μL reactions contained 10 μL of SYBR Premix Ex Taq (TaKaRa), 0.2 µM of each primer, 2 μL of template DNA, and 7.2 μL of ddH_2_O. The reaction program was set as follows: initial denaturation at 95 °C for 30 s, 40 cycles at 95 °C for 5 s, annealing temperature for 30 s and 72 °C for 30 s, then a melt curve stage with temperature ramping from 60 °C to 95 °C.

### 2.7. Statistical Analysis

Ten plant samples from each treatment were used to test the effect of antibiotics on plant growth. For other analyses, three repetitions, each of which was the mixture of different plant parts from six plants, were performed. The mean values and standard deviations (SDs) of all data were calculated using Microsoft Office Excel 2016 (Microsoft, Redmond, WA, USA). Statistical analyses were performed using the software SPSS 21.0 (IBM, Armonk, NY, USA). Duncan’s multiple comparisons were used to determine the significant differences (*p* < 0.05) between treatments.

## 3. Results

### 3.1. Effects of Antibiotic Exposure on Pakchoi Growth

**Figure 1:**
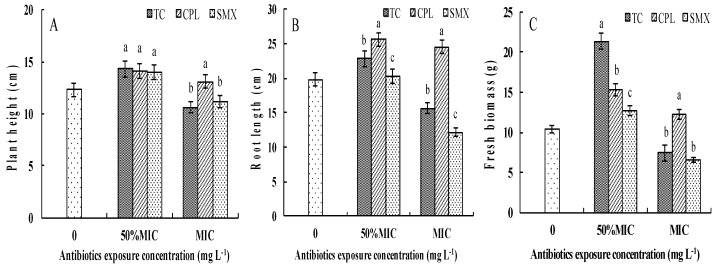
Effects of different dosages and types of antibiotic exposure on the growth of pakchoi under hydroponic condition. (**A**) Plant height; (**B**) Root length; (**C**) Fresh biomass. Values are mean ± SD (*n* = 10). Different letters on the top of the error bars indicate statistically difference among the treatments (*p* < 0.05). TC, tetracycline; CPL, cephalexin; SMX, sulfamethoxazole; MIC, minimum inhibitory concentration; SD, standard deviation.

The effects of antibiotics on pakchoi growth were assessed by analyzing the growth parameters. The detected values of plant height, root length, and fresh biomass are shown in Table S4. Figure 1 shows the changes of pakchoi growth at different antibiotic types and doses. Compared with the control, the growth indices increased when exposed to 50% MIC levels of antibiotics (*p* < 0.0001 for the plant heights and *p* < 0.001 for the fresh biomass values). When the exposure dose was increased to MIC levels, this growth-promoting effect was maintained in CPL-treated plants. However, the TC- and SMX-treated plants were significantly inhibited (*p* < 0.01), as indicated by the growth parameters being less than those of the controls. According to the detection of growth inhibition rates (data shown in Table S4), fresh plant biomass was the most affected parameter (fresh biomass > root length > plant height) under antibiotic exposure. SMX showed the greatest impact on pakchoi growth in the three antibiotic types and CPL had the least inhibition on the plants.

### 3.2. Antibiotic Uptake by Pakchoi

**Figure 2:**
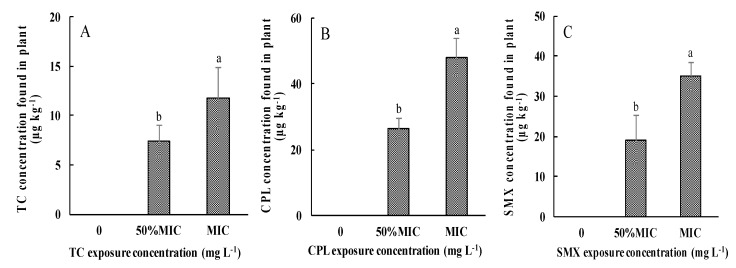
Accumulation of antibiotics in hydroponic pakchoi under different dosages of antibiotic exposure. (**A**) TC exposure treatment; (**B**) CPL exposure treatment; (**C**) SMX exposure treatment. Values are mean ± SD (*n* = 3). Different letters on the top of the error bars indicate statistical difference among the treatments (*p* < 0.05).

The edible portions of pakchoi samples, both controls and antibiotic-exposed plants, were separated and the concentrations of TC, CPL, and SMX within plants were determined to evaluate the uptake of antibiotics by plants, as shown in Figure 2. The results indicated a concentration range from 6.9 to 11.8 µg·kg^−1^ for TC (Figure 2A), 26.4 to 48.1 µg·kg^−1^ for CPL (Figure 2B), and 18.1 to 35.3 µg·kg^−1^ for SMX (Figure 2C) in plants, respectively. However, no antibiotic accumulation was detected in the controls. Obviously, the antibiotic concentrations in the vegetables increased as the antibiotic dose increased in the culture solution. The mean CPL concentration in the CPL-treated samples was higher than those of the other antibiotic residuals in their corresponding treated samples when the exposure concentrations were at MIC levels.

### 3.3. Effects of Antibiotic Exposure on AREB in Pakchoi

**Figure 3:**
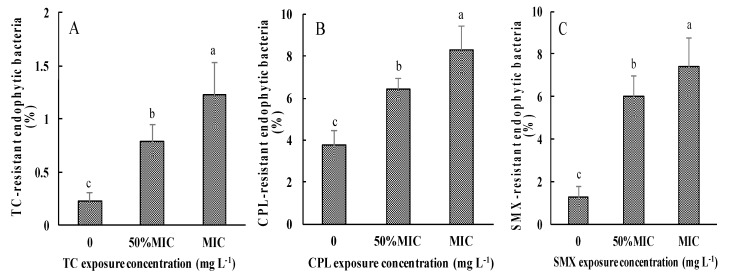
Rates of AREB to TCEB in pakchoi under different dosages of antibiotic exposure. (**A**) TC exposure treatment; (**B**) CPL exposure treatment; (**C**) SMX exposure treatment. Values are mean ± SD (*n* = 3). Different letters on the top of the error bars indicate statistical difference among the treatments (*p* < 0.05).

The CFUs of TCEB and AREB in plant tissues under different antibiotic treatment conditions were determined, and the rates of AREB to TCEB were calculated, as shown in Figure 3. Compared with the controls, the cultivable AREB levels in TC-, CPL-, and SMX-treated samples reached 0.61–0.85 × 10^3^, 4.63–5.36 × 10^3^, and 4.89–5.18 × 10^3^ CFU·g^−1^, respectively, which were higher than those in the control samples (0.23 × 10^3^, 3.77 × 10^3^, and 1.26 × 10^3^ CFU·g^−1^). These changes in AREB abundance resulted in dramatic increases in the ratios of AREB to TCEB from 0.23%, 3.77%, and 1.26% of TC, CPL, and SMX resistance, respectively, in the controls, to 0.79–1.23% (Figure 3A), 6.41–8.29% (Figure 3B), and 6.00–7.43% (Figure 3C), respectively, in the corresponding antibiotic-treated plants.

### 3.4. Abundance of ARGs in the Pakchoi Endophytic System

**Figure 4:**
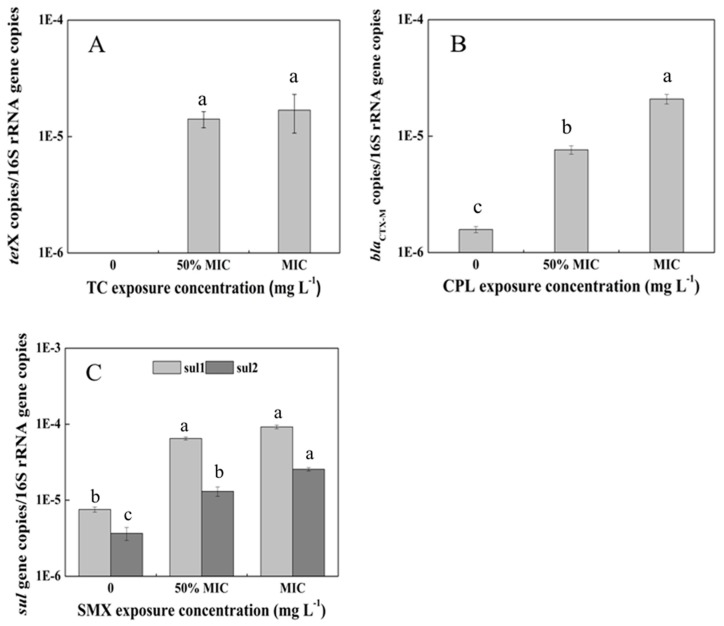
Abundance of antibiotic resistance genes (ARGs) in the endophytic system of pakchoi under different dosages of antibiotic exposure. (**A**) *tet*X gene in TC-treated plants; (**B**) *bla*_CTX-M_ gene in CPL-treated plants; (**C**) *sul*1 and *sul*2 genes in SMX-treated plants. Values are mean ± SD (*n* = 3). Different letters on the top of the error bars indicate statistical difference among the treatments (*p* < 0.05).

In total, 23 ARGs corresponding to three antibiotics were detected in antibiotic-treated vegetables using the PCR technique. Among them, only one *tet* gene (*tet*X), one β-lactamase gene (*bla*_CTX-M_), and two sul genes (*sul*1 and *sul*2) responsible for TC, CPL, and SMX resistance, respectively, were present in the corresponding antibiotic-treated samples. Thus, further quantification using qPCR was conducted to monitor their responses to different treatment doses. To minimize the differences in background bacterial abundances and DNA extraction efficiency, 16S rRNA gene was also quantified and the absolute numbers of the above four quantified ARGs were normalized to that of the ambient 16S rDNA (Figure 4). In the control plants without antibiotic treatment, the values of the *tet*X gene were under the detection limit but the other three ARGs were detected at ~10^−6^ copies/16S rRNA gene copies. For the *tet*X gene, the relative abundance continuously increased as the TC dose increased. Sul and bla genes, on the whole, showed similar changes during the planting period. The four ARGs all reached their highest relative abundances of 10^−5^ to 10^−4^ copies/16S rRNA gene copies at the MIC exposure levels, which were one to two orders of magnitude greater than those in the control samples. Thus, the variation trends of the *tet*X, *sul*1, *sul*2, and *bla*_CTX-M_ genes during different antibiotic treatments demonstrated great approximations.

## 4. Discussion

Plants are an important component of terrestrial ecosystems and are a potential pathway for antibiotic transport because of their absorption capacity [33]. Our previous studies indicated that cephalosporin, tetracyclines, and sulfonamides were the most frequently used antimicrobial agents in livestock farms in China, and high ratios of AREB occurred in the livestock manure fertilized field vegetables [28]. The transformation of these antibiotics and their induction of antibiotic resistance in soil or water environments have been frequently reported [6,41,42]. Further study through pot planting experiments confirmed different compositions of AREB presence in vegetable endophytic systems [43]. Therefore, TC, CPL, and SMX were selected to explore their accumulation and induction in pakchoi endophytic systems in this study. To simplify the conditions, a series of hydroponic cultures of pakchoi were used. Based on the Clinical and Laboratory Standards Institute (CLSI) standards [32] and the bacterial community composition in the pakchoi endophytic system [43], the highest MIC values of TC, CPL, and SMX for different genera of endophytic bacteria were selected and set as the levels of antibiotic exposure in the present study. From our results, we can see that although the exposure doses of different drugs were greater than their practical occurrence in agroecosystems, the pakchoi still showed natural growth throughout the planting process. Moreover, according to our previous investigation [28], sometimes animal manure containing high concentrations of antibiotic residue also will be used for field plants. Therefore, this study provided direct evidence for the effects of antibiotics on plant growth and the development of antibiotic resistance, especially under different treatment doses. The changes in growth parameters indicated the phytotoxic levels of each antibiotic at different doses. Generally, at 50% MIC levels, antibiotics stimulated growth, increasing plant fresh biomass production. However, at MIC levels, the antibiotics acted as inhibitors, reducing yields and inducing metabolic disturbances. Previous research had indicated that low antibiotic concentrations are beneficial for plant growth, whereas high antibiotic concentrations can induce toxicity [44]. In a comparison of the three antibiotics, CPL has the lowest toxicity to pakchoi. This may be due to β-lactam’s specific actions on bacterial cell wall components, which are targets that do not exist in plant cells [45]. Therefore, the growth inhibition rate was lowest under CPL exposure when compared with controls.

The detection of the three antibiotics in pakchoi tissues indicates the uptake and transfer of antibiotics from the water environment to the vegetable, which is similar to previous results [14,18]. The bioaccumulation of antibiotics in plants can vary depending on plant species and antibiotic class [15,20]. Usually, ionization, as well as the properties of sorption and water solubility, can directly affect how plants uptake pharmaceuticals [21]. CPL was noted to have accumulated to the highest concentration among the study compounds at the MIC exposure level, which may reflect the greater absorbency of CPL compared with the other compounds. Furthermore, the concentrations of antibiotics in pakchoi did not increase unlimitedly as antibiotic dose increased. The probable reasons include: (1) the saturation level of antibiotic accumulation was reached; (2) the incorporated antibiotics were stored in the plant cells, in which they can be degraded; and (3) the degradation of antibiotics was accelerated by the release of plant enzymes during sample grinding [20]. Nevertheless, vegetables that have accumulated antibiotics from contaminated environments will be consumed by humans, and then might be absorbed by the human body, resulting in increased antibiotic resistance, including the emergence of multidrug-resistant bacteria, which leads to antibiotic treatment failures [13,46].

Few studies have explored the influence of antibiotic pollution on endophytic systems in vegetables. Yet vegetables contaminated by antibiotics may contribute to the development of AREB. The present study showed that the rates of AREB occurrence and the relative abundances of ARGs increased in pakchoi endophytic systems after exposure to the three antibiotics. The trends of these changes were comparable with the results from our previous study, in which a distinct increase in some AREBs was shown in manure- or organic fertilizer-amended pakchoi samples [43]. Two possible factors may contribute to such increases. Firstly, during the pakchoi cultivation, there would be a natural rhizosphere microbial consortium forming in the nutrient solution. However, spiked antibiotics as a selective pressure could influence the microbial community compositions and induce the occurrence of high ratios of resistance. This has been proved in many other environments even at much lower concentrations than their MIC values [47,48,49,50]. Thus, a special microbial consortium would be established corresponding to different treatments of antimicrobial agents. As we know, environmental bacteria, especially rhizosphere bacteria, are an important source for plant endophytic bacteria [51], which could enter through the tissues to the plant endophytic systems, thus resulting in high occurrence of AREB in the plant. Secondly, the AREB can be persistent in the plant endophytic systems. In the present study, the accumulated antibiotics in plants, although below their corresponding minimum MIC values for various species of bacteria, might also provide a selection pressure to the endophytic bacteria, thereby providing the AREB survival advantages in the polluted environments. The qPCR also demonstrated that the abundance of ARGs in the endophytic bacteria corresponding to the three antibiotics continuously increased as the antibiotic uptake in the pakchoi increased.

Among the four detected ARGs, the presence of *tet*X, a special enzymatic modification gene for the degradation of TCs, may be related to the low detected TC concentration in pakchoi. In addition, the absence of the *tet*X gene in control samples and the persistence of it in the treated samples may indicate the potential transfer of bacteria carrying the *tet*X gene from the environment to plant. Notably, the third-generation drug tigecycline has been used in clinical treatments due to its broad spectrum of antibacterial activity (especially inhibiting multiple antibiotic-resistant bacteria and super bacteria) [52,53,54]. However, the bacterial strains containing the *tet*X gene isolated from patients are still resistant to tigecycline [55]. Therefore, the prevalence of the *tet*X gene in edible pakchoi should be highly concerning. The other ARGs, *sul*1, *sul*2, and *bla*_CTX-M_, are widely present in various environmental media [56,57,58], and act as the most prevalent mechanisms of sulfonamide and β-lactam resistance, respectively. In particular, the *sul*1 gene is normally found linked to other resistance genes in the Tn21 type integron, while *sul*2 is usually located on small plasmids of the IncQ family [59]. qPCR showed that the abundance levels of these ARGs increased in antibiotic contaminated environments, indicating their enrichment and transmission under antibiotic selection pressure.

Previous studies have demonstrated the antibiotic uptake [22] and the presence of resistant human pathogens or opportunistic pathogens in vegetables planted in manure-amended soil [43]. Thus, accumulated antibiotics in vegetables and the prevalence of antibiotic resistance in endophytic systems might be disseminated to humans when these vegetables are consumed. Consequently, evaluating the biological responses of terrestrial crops to antibiotics, especially frequently consumed vegetables, is important. However, compared with the soil environment, this hydroponic cultivation system is just a simple model to evaluate the influences of antibiotics on plant growth and plant endophytic bacteria. Further research is required to study the community compositions of AREB corresponding to different types of antibiotic exposure under soil cultivation systems. The results will provide basic information for an integrative risk assessment of antibiotic application and food security.

## 5. Conclusions

The present study investigated the growth of pakchoi and the antibiotic resistance in its endophytic system under TC, CPL, and SMX exposure. Pakchoi was shown to absorb antibiotics from the hydroponic culture environment. The absorption was selective toward different antibiotics, and the absorption amount was related to the antibiotic concentration. The accumulated antibiotics in the plant influenced the growth of the plant and increased the levels of AREB and ARGs, even at sub-inhibitory doses, which should be noted due to considerations surrounding the possible transfer of ARGs through the food chain.

## Supplementary Materials

The following are available online at www.mdpi.com/1660-4601/14/11/1336/s1, Table S1: PCR primers, annealing temperatures, and resistance mechanisms, Table S2: qPCR primers and annealing temperatures used in the present study, Table S3: qPCR standard curves for 16S rRNA gene and antibiotic resistance genes, Table S4: Root length, plant height, and fresh biomass values of hydroponic pakchoi under different dosages of antibiotic treatment.
