# Supplementary material for: Plant Growth, Antibiotic Uptake, and Prevalence of Antibiotic Resistance in an Endophytic System of Pakchoi under Antibiotic Exposure
Source: Int J Environ Res Public Health. 2017 Nov 3;14(11):1336. doi: 10.3390/ijerph14111336 (PMC5707975; doi:10.3390/ijerph14111336)
Supplement: Supplementary file 1 [file ijerph-14-01336-s001.pdf]

Supplementary Material

Plant Growth, Antibiotic Uptake, and Prevalence of Antibiotic Resistance in an Endophytic System of Pakchoi under Antibiotic Exposure

Hao Zhang, Xunan Li, Qingxiang Yang, Linlin Sun, Xinxin Yang, Mingming Zhou, Rongzhen Deng, and Linqian Bi

Table S1–S3

Table S1. PCR primers, annealing temperatures, and resistance mechanisms

Table S2. qPCR primers and annealing temperatures used in the present study

Table S3. qPCR standard curves for 16S rRNA gene and antibiotic resistance genes

Table S4. Root length, plant height, and fresh biomass values of hydroponic pakchoi under different dosages of antibiotic treatment

Table S1. PCR primers, annealing temperatures, and resistance mechanisms.

Gene name	Primer	5′–3′ sequence	Resistance Mechanism	Anneal temp. (°C)	Reference
*tet*A	FW	GCTACATCCTGCTTGCCTTC	efflux pump	55	[1]
	RV	CATAGATCGCCGTGAAGAGG			
*tet*C	FW	CTTGAGAGCCTTCAACCCAG	efflux pump	55	[1]
	RV	ATGGTCGTCATCTACCTGCC			
*tet*G	FW	GCTCGGTGGTATCTCTGCTC	efflux pump	55	[1]
	RV	AGCAACAGAATCGGGAACAC			
*tet*K	FW	CGAAAACAGACTCGCCAATC	efflux pump	55	[1]
	RV	TCCATAATGAGGTGGGGC			
*tet*L	FW	TCGTTAGCGTGCTGTCATTC	efflux pump	55	[1]
	RV	GTATCCCACCAATGTAGCCG			
*tet*M	FW	ACAGAAAGCTTATTATATAAC	ribosomal protection protein	45	[2]
	RV	TGGCGTGTCTATGATGTTCAC			
*tet*O	FW	ACGGARAGTTTATTGTATACC	ribosomal protection protein	45	[2]
	RV	TGGCGTATCTATAATGTTGAC			
*tet*Q	FW	AGAATCTGCTGTTTGCCAGTG	ribosomal protection protein	55	[2]
	RV	CGGAGTGTCAATGATATTGCA			
*tet*T	FW	AAGGTTTATTATATAAAAGTG	ribosomal protection protein	45	[2]
	RV	AGGTGTATCTATGATATTTAC			
*tet*W	FW	GAGAGCCTGCTATATGCCAG	ribosomal protection protein	55	[2]
	RV	GGGCGTATCCACAATGTTAAC			
*tet*X	FW	CAATAATTGGTGGTGGACCC	enzymatic modification	55	[1]
	RV	TTCTTACCTTGGACATCCCG			
*tet*B/P	FW	AAAACTTATTATATTATAGTG	ribosomal protection protein	45	[3]

	RV	TGGAGTATCAATAATATTCAC			
sul1	FW	CGGCGTGGGCTACCTGAACG	ribosomal protection protein	60	[2]
	RV	GCCGATCGCGTGAAGTTCCG			
*sul*2	FW	GCGCTCAAGGCAGATGGCATT	ribosomal protection protein	60	[4]
	RV	GCGTTTGATACCGGCACCCGT			
*sul*3	FW	TCAAAGCAAAATGATATGAGC	ribosomal protection protein	50	[5]
	RV	TTTCAAGGCATCTGATAAAGAC			
*dfr*A1	FW	AGCATTACCCAACCGAAAGT	enzymatic modification	50	[6]
	RV	TGTCAGCAAGATAGCCAGAT			
*dfr*A7	FW	AAATGGCGTAATCGGTAATG	enzymatic modification	50	[6]
	RV	GTGAACAGTAGACAAATGAAT			
bla_{ampC}	FW	TGGCGTATCGGGTCAATGT	enzymatic modification	55	[5]
	RV	CTCCACGGGCCAGTTGAG			
bla_{VIM}	FW	GCACTTCTCGCGGAGATTG	enzymatic modification	55	[5]
	RV	CGACGGTGATGCGTACGTT			
$bla_{CTX\text{-}M}$	FW	ATGTGCAGYACCAGTAARGTKATGGC	enzymatic modification	55	[7]
	RV	ATCACKCGGRTCGCCNGGRAT			
bla_{TEM}	FW	TCGGGGAAATGTGCG	enzymatic modification	50	[7]
	RV	GGAATAAGGGCGACA			
bla_{SHV}	FW	CTTTCCCATGATGAGCACCTTT	enzymatic modification	55	[5]
	RV	TCCTGCTGGCGATAGTGGAT			
bla_{Z}	FW	GGAGATAAAGTAACAAATCCAGTTAGATATGA	enzymatic modification	55	[5]
	RV	TGCTTAATTTTCCATTTGCGATAAG			

Table S2. qPCR primers and annealing temperatures used in the present study.

Gene name	Primer	5′–3′ sequence	Product size (bp)	Anneal temp. (°C)	Reference
16S rRNA	338F	CCTACGGGAGGCAGCAG	202	60	[8]
	518R	ATTACCGCGGCTGCTGG			
*tet*X	FW	AGCCTTACCAATGGGTGTAAA	278	60	[9]
	RV	TTCTTACCTTGGACATCCCG			
sul1	FW	CCGTTGGCCTTCCTGTAAAG	67	60	[10]
	RV	TTGCCGATCGCGTGAAGT			
*sul*2	FW	CTCCGATGGAGGCCGGTAT	190	60	[10]
	RV	GGGAATGCCATCTGCCTTGA			
bla_{CTX-M}	FW	GGAGGCGTGACGGCTTTT	101	51	[5]
	RV	TTCAGTGCGATCCAGACGAA			

Table S3. qPCR standard curves for 16S rRNA gene and antibiotic resistance genes.

Gene name	Standard curves	R^2
16S rRNA	Y = −3.407 × Log(X) + 36.004	0.99497
*tet*X	Y = −3.0462 × Log(X) + 34.639	0.99532
sul1	Y = −2.9326 × Log(X) + 34.148	0.99082
*sul*2	Y = −3.425 × Log(X) + 36.19	0.99686
bla_{CTX-M}	Y = −2.7213 × Log(X) + 33.879	0.99391

Table S4. Root length, plant height, and fresh biomass values of hydroponic pakchoi under different dosages of antibiotic treatment.

Antibiotic concentration (mg L^{-1})	Root length		Plant height		Fresh biomass	
	average value (cm)	inhibition rate (%)	average value (cm)	inhibition rate (%)	average value (g)	inhibition rate (%)
**TC treatment**						
0	19.8 ± 3.12 b*		12.45 ± 1.14 b		10.72 ± 2.46 b	
50%MIC	22.8 ± 2.44 a	−15.15	13.85 ± 1.06 a	−11.24	20.75 ± 3.09 a	−93.53
MIC	15.6 ± 1.51 c	21.21	10.8 ± 1.23 c	13.25	8.01 ± 1.99 c	25.31
**CPL treatments**						
0	19.8 ± 3.12 b		12.45 ± 1.14 bc		10.72 ± 2.46 b	
50%MIC	25.6 ± 2.79 a	−29.29	14. 5 ± 2.07 a	−16.47	15.06 ± 2.90 a	−40.44
MIC	24.5 ± 3.24 a	−23.73	13.1 ± 1.29 b	−5.22	12.97 ± 1.87 a	−20.99
**SMX treatments**						
0	19.8 ± 3.12 a		12.45 ± 1.14 b		10.72 ± 2.46 b	
50%MIC	20.3 ± 0.95 a	−2.53	13.95 ± 1.17 a	−12.05	12.73 ± 1.47 a	−18.72
MIC	12.05 ± 2.52 b	39.14	11.15 ± 1.20 c	10.44	6.51 ± 1.53 c	39.28

The values are mean ± SD (n = 10). * Different letters indicate the differences in the value among the treatments based on the LSD (Least Significant Difference) test ($P < 0.05$)

Commented [Edit1]: Consider defining.

References

1. Ng, L.K.; Martin, I.; Alfa, M.; Mulvey, M. Multiplex PCR for the detection of tetracycline resistant genes. *Mol. Cell Probes* **2001**, *15*, 209–215.
2. Aminov, R.I.; Garrigues-Jeanjean, N.; Mackie, R.I. Molecular ecology of tetracycline resistance: development and validation of primers for detection of tetracycline resistance genes encoding ribosomal protection proteins. *Appl. Environ. Microbiol.* **2001,** *67*, 22–32.
3. Mu, Q.; Li, J.; Sun, Y.; Mao, D.; Wang, Q.; Yi, L. Occurrence of sulfonamide-, tetracycline-, plasmid-mediated quinolone- and macrolide-resistance genes in livestock feedlots in northern China. *Environ. Sci. Pollut. Res.* **2015**, *22*, 6932–6940.
4. Kerrn, M.B.; Klemmensen, T.; Frimodt-Møller, N.; Espersen, F. Susceptibility of danish *Escherichia coli* strains isolated from urinary tract infections and bacteraemia, and distribution of *sul* genes conferring sulphonamide resistance. *J. Antimicrob. Chemother.* **2002**, *50*, 513–516.
5. Zhu, Y.G.; Johnson, T.A.; Su, J.Q.; Qiao, M.; Guo, G.X.; Stedtfeld, R.D.; Hashsham, S.A.; Tiedje, J.M. Diverse and abundant antibiotic resistance genes in Chinese swine farms. *Proc. Natl. Acad. Sci. USA* **2013**, *110*, 3435–3440.
6. Selvam, A.; Xu, D.L.; Zhao, Z.Y.; Wong, J.W.C. Fate of tetracycline, sulfonamide and fluoroquinolone resistance genes and the changes in bacterial diversity during composting of swine manure. *Bioresour. Technol.* **2012**, *126*, 383–390.
7. Knapp, C.W.; Zhang, W.; Sturm, B.S.M.; Graham, D.W. Differential fate of erythromycin and beta-lactam resistance genes from swine lagoon waste under different aquatic conditions. *Environ. Pollut.* **2010**, *158*, 1506–1512.
8. Muyzer, G.; De Waal, E.C.; Uitterlinden, A.G. Profiling of complex microbial populations by denaturing gradient gel electrophoresis analysis of polymerase chain reaction-amplified genes coding for 16S rRNA. *Appl. Environ. Microbiol.* **1993**, *59*, 695-700.
9. Ghosh, S.; Ramsden, S.J.; LaPara, T.M. The role of anaerobic digestion in controlling the release of tetracycline resistance genes and class 1 integrons from municipal wastewater treatment plants. *Appl. Microbiol. Biot.* **2009**, *84*, 791–796.
10. Heuer, H.; Smalla, K. Manure and sulfadiazine synergistically increased bacterial antibiotic resistance in soil over at least two months. *Environ. Microbiol.* **2007**, *9*, 657–666.
